# Optimized reconstruction of undersampled Dixon sequences using new memory‐efficient unrolled deep neural networks: HalfVarNet and HalfDIRCN

**DOI:** 10.1002/mrm.70070

**Published:** 2025-09-08

**Authors:** Sandra Martin, Amira Trabelsi, Maxime Guye, Marc Dubois, Redha Abdeddaim, David Bendahan, Rémi André

**Affiliations:** ^1^ Multiwave Technologies SAS Marseille France; ^2^ Aix Marseille Univ, CNRS, CRMBM Marseille France; ^3^ Aix Marseille Univ, CNRS, Centrale Med Institut Fresnel Marseille France

**Keywords:** biomarker, memory‐efficient learning, MRI acceleration, neuromuscular disorders, unrolled reconstruction, Variational network

## Abstract

**Purpose:**

Fat fraction (FF) quantification in individual muscles using quantitative MRI is of major importance for monitoring disease progression and assessing disease severity in neuromuscular diseases. Undersampling of MRI acquisitions is commonly used to reduce scanning time. The present paper introduces novel unrolled neural networks for the reconstruction of undersampled MRI acquisitions. These networks are designed with the aim of maintaining accurate FF quantification while reducing reconstruction time and memory usage.

**Methods:**

The proposed approach relies on a combination of a simplified architecture (Half U‐Net) with unrolled networks that achieved high performance in the well‐known FastMRI challenge (variational network [VarNet] and densely interconnected residual cascading network [DIRCN]). The algorithms were trained and evaluated using 3D MRI Dixon acquisitions of the thigh from controls and patients with neuromuscular diseases. The study was performed by applying a retrospective undersampling with acceleration factors of 4 and 8. Reconstructed images were used to computed FF maps.

**Results:**

Results disclose that the novel unrolled neural networks were able to maintain reconstruction, biomarker assessment, and segmentation quality while reducing memory usage by 24% to 16% and reducing reconstruction time from 21% to 17%. Using an acceleration factor of 8, the proposed algorithms, HalfVarNet and HalfDIRCN, achieved structural similarity index (SSIM) scores of 93.76 ± 0.38 and 94.95 ± 0.32, mean squared error (MSE) values of 12.76 ± 1.08 × 10^−2^ and 10.25 ± 0.87 × 10^−2^, and a relative FF quadratic error of 0.23 ± 0.02% and 0.17 ± 0.02%, respectively.

**Conclusion:**

The proposed method enables time and memory‐efficient reconstruction of undersampled 3D MRI data, supporting its potential for clinical application.

## 1 INTRODUCTION

MRI is a powerful non‐invasive imaging modality offering strong soft tissue contrast. Despite its numerous advantages, MRI suffers from long acquisition time. Reducing scan time enables to enhance patient comfort, saves healthcare costs, and minimizes motion artifacts in the MRI images.

MRI data are conventionally acquired in the spatial frequency domain (k‐space), and the fully sampled data can be directly reconstructed using the inverse Fourier transform. Common strategies to accelerate MRI acquisitions, such as parallel imaging^1^, ^2^ and compressed sensing,^3^ consist in dropping information in the k‐space. Parallel imaging requires regular undersampling with cartesian acquisition yielding coherent aliasing in the image domain. Missing data can be recovered using information redundancy from a multi‐coil array. Image can be restored either by removing coherent aliasing in the image domain^2^ or by interpolating missing frequencies in the k‐space.^1^ Irregular undersampling and/or non‐cartesian (spiral,^4^ radial,^5^ variational density,^6^ or sparkling^7^) acquisitions yield incoherent aliasing (noise‐like). Compressed sensing enables reconstruction of apparently noise free images by ensuring consistency with k‐space measurements and by enforcing sparsity in a known transform domain. Formally, compressed sensing is an optimization problem under sparsity constraint. Solving the latter optimization problem requires sophisticated iterative algorithms which aim at minimizing both data consistency and regularization terms^8^ (Eq. 2 shows a basic optimizer for compressed sensing). Despite their effectiveness, these algorithms face limitations such as long reconstruction time or non‐trivial hyperparameters tuning.^9^ A poor choice of hyperparameters can lead either to significant residual undersampling artifacts or unrealistic structures in the reconstructed images. Khare et al.^10^ proposed a data‐driven approach for the automatic selection of hyperparameters. However, the choice of the sparsifying transform domain in the regularization term strongly depends on the type of images. For instance, images from MR angiography are directly sparse in the image domain. Total variation can be used when images are piecewise constant. Wavelet domain is commonly used since most natural images can be sparsely represented. From a clinical point of view, classical sparsifying transform domains may not be able to capture complex structures associated with biological tissues. This issue was addressed by learning the transform domain through dictionary learning techniques.^11^ However, image reconstruction relying on iterative algorithms remained time‐consuming.

**FIGURE 1:**
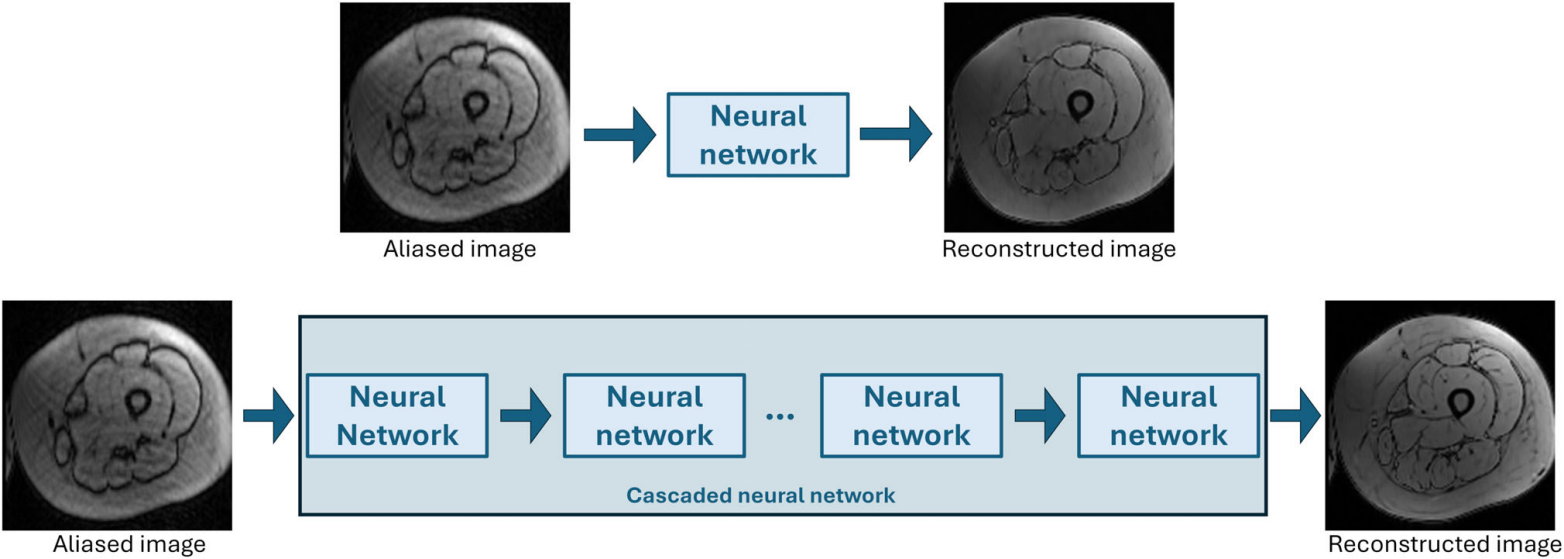
(Top) A typical neural network that links an input to an output in a single pass. (Bottom) A cascaded neural network architecture. Multiple neural networks follow one another sequentially. The output of each network becomes the input of the next enabling progressive refinement.

Deep learning methods have shown promising results in the MRI reconstruction field.^12^, ^13^ Particularly, unrolled neural networks have shown great success. The main idea is based on the unrolling of the iterations of classical reconstruction algorithms. An iteration is then modeled as a block composed of a data consistency update and a neural network acting as a regularizer update. An unrolled neural network can be seen as cascaded networks (Figure 1). Hammernik et al.^14^ proposed a variational network using a simple convolutional neural network as regularizer update, based on field of experts models (FoE models).^15^ Improvements to variational network (VarNet) led to the development of end‐to‐end VarNet (E2E VarNet),^16^ by replacing FoE models with U‐Net.^17^ Densely interconnected residual cascading network (DIRCN)^18^ was inspired by VarNet and incorporates dense connections between cascades. Recurrent VarNet^19^ adapts VarNet using recurrent unit instead of U‐Net. However, using a recurrent network instead of U‐Net requires more memory to store the gradient during training. Giannakopoulos et al.^20^ introduced Feature VarNet to enable training in the feature space. Finally, Feature VarNet was combined with the traditional image‐space approach (E2E VarNet) to create Feature Image VarNet with the goal of improving performance. Of interest, self‐supervised learning, which does not require fully sampled data for training step, is another promising strategy for MRI reconstruction methods.^21^ While numerous unrolled networks have been proposed,^12^ most of them deal with 2D data only and/or are limited by the number of cascades. This limitation is strongly accentuated in the case of 3D MRI acquisitions. Handling the 3D structure directly impacts computational cost and leads to a dramatic reconstruction time increase. For example, switching from 2D to 3D data represents a training time three times longer. Moreover, the storage of high dimensional tensors during the training may make the computation of gradients via backpropagation infeasible due to memory limitations of graphics processing unit (GPU). Wang et al.^22^ use a memory efficient learning framework, introduced by Kellman et al.^23^ to address the GPU memory limitation but increase the training time. Miller et al.^24^ use another memory efficient model based deep learning for non‐cartesian 3D data. Comparison with a classical compressed sensing approach showed that training time was reduced while improving image quality.

MRI has been extensively used for the diagnosis and follow‐up of patients with neuromuscular disorders (NMD). NMD are rare diseases characterized by progressive muscle weakness and loss of motor skills which is mainly related to a fibro‐adipotic process resulting in the replacement of muscle tissue by fat.^25^ Of interest, this replacement can be monitored by quantitative MRI and more particularly from the Dixon‐based fat fraction (FF) measurement. Accordingly, FF has been acknowledged as a sensitive biomarker of disease progression.^26^ FF maps are obtained using Dixon acquisitions which enable fat‐water separation.^27^ The corresponding fat and water maps can be combined to compute FF values at the voxel level. In 2014, Hollingsworth et al.^28^ proposed a rigorous comparative study between parallel imaging and compressed sensing for the acceleration of Dixon sequences and the follow‐up of NMD. Since quantifying FF in each individual muscle of the thigh is of major importance, it is necessary to be able to delineate each of them. The performance was then evaluated on reconstruction metrics, FF assessment, and capability of experts to segment reconstructed images. The latter study concluded that parallel imaging and compressed sensing can be combined (using eSPIRIT^29^) to accelerate FF measurement.

In the present work, we propose two novel memory‐efficient and time‐efficient learning methods: HalfVarNet and HalfDIRCN. The principle is to replace U‐Net modules in VarNet and DIRCN by Half U‐Net.^30^ Half U‐Net has been previously proposed in 2D segmentation to reduce the number of parameters and the memory use while achieving comparable segmentation results. However, it has not yet been used in MRI reconstruction. Our experimentations follow the pattern proposed Hollingsworth et al.^28^ Experts performing the segmentation task are here replaced by nnU‐Net,^31^ an efficient segmentation neural network inspired by U‐Net which was trained on fully sampled images. In all situations, results clearly show the benefits of HalfVarNet and HalfDIRCN in comparison with VarNet and DIRCN. The proposed approach allows two interesting options. Either we use the same number of cascades as VarNet and DIRCN or we increase the number of cascades. The first option leads to save GPU memory while maintaining the performance. The second option does not allow to save GPU memory but outperforms reconstruction performance. In summary, our main contributions are (i) the design and development of memory‐efficient and fast unrolled neural networks for 3D MRI reconstruction: HalfVarNet and HalfDIRCN; and (ii) experimental results from a realistic biomedical application with 3D MRI acquisitions, namely the follow‐up of patients suffering from neuromuscular diseases.

## 2 METHODS

### 2.1 Problem formulation

Let *k* be the complex k‐space values to be reconstructed and $k_{\mathrm{us}}$ the undersampled k‐space measurement. We aim to reconstruct k from $k_{\mathrm{us}}$ by solving the following optimization problem:

(1) $$\hat{k}=\underset{k}{\text{argmin}}\;{\left\|k_{\mathrm{us}}-\mathrm{Dk}\right\|}_{2}^{2}+\lambda R\left(F^{H}k\right)$$

where *D* is an undersampling binary mask, *F* is the Fourier transform, *F* ^*H*^ is the inverse Fourier transform, and λ is a penalization parameter. The first term enforces data consistency with undersampled measurement $k_{\mathrm{us}}$, while R is a regularization function. Equation (1) can be solved using iterative optimization algorithms. Assuming *R* is differentiable, an iteration of gradient descent algorithm yields

(2) $$\begin{array}{c} {\hat{k}}_{\mathrm{it}+1}={\hat{k}}_{\mathrm{it}}-\mu_{\mathrm{it}}\left(D\left({\hat{k}}_{\mathrm{it}}-k_{\mathrm{us}}\right)+\lambda F\nabla_{k}R\left(F^{H}{\hat{k}}_{\mathrm{it}}\right)\right) \end{array}$$

where $\mu_{\mathrm{it}}$ is the gradient descent step of the current iteration *it*, and $\nabla_{k}R$ is the gradient of R with respect to *k*. The initial guess ${\hat{k}}_{1}$ is often chosen as $k_{\mathrm{us}}$ where the non‐acquired k‐space points are set to 0.

### 2.2 Unrolled networks

MRI reconstruction from undersampled data is an inherently ill‐posed problem. Consequently, the choice of R is crucial to reconstruct realistic images. Compressed sensing is based on the prior assumption that images are sparse in certain transform domains. Classical sparsifying transform domains are often too simple to capture complex structures associated with biological tissues. Additionally, regularization techniques employed in compressed sensing require manual selection of the penalization parameters λ potentially yielding noisy or unrealistic images. To overcome these limitations, convolutional neural networks (CNN) can be used to learn the gradient of the regularization function $\nabla_{k}R$ as well as $\mu_{\mathrm{it}}$ directly from the data. These networks, called unrolled networks, mimic the gradient descent scheme. The present work focuses on VarNet^16^ and DIRCN.^18^ This choice was motivated because both networks achieved the third and fourth place in the *fastMRI* challenge.^20^

#### 2.2.1 VarNet

**FIGURE 2:**
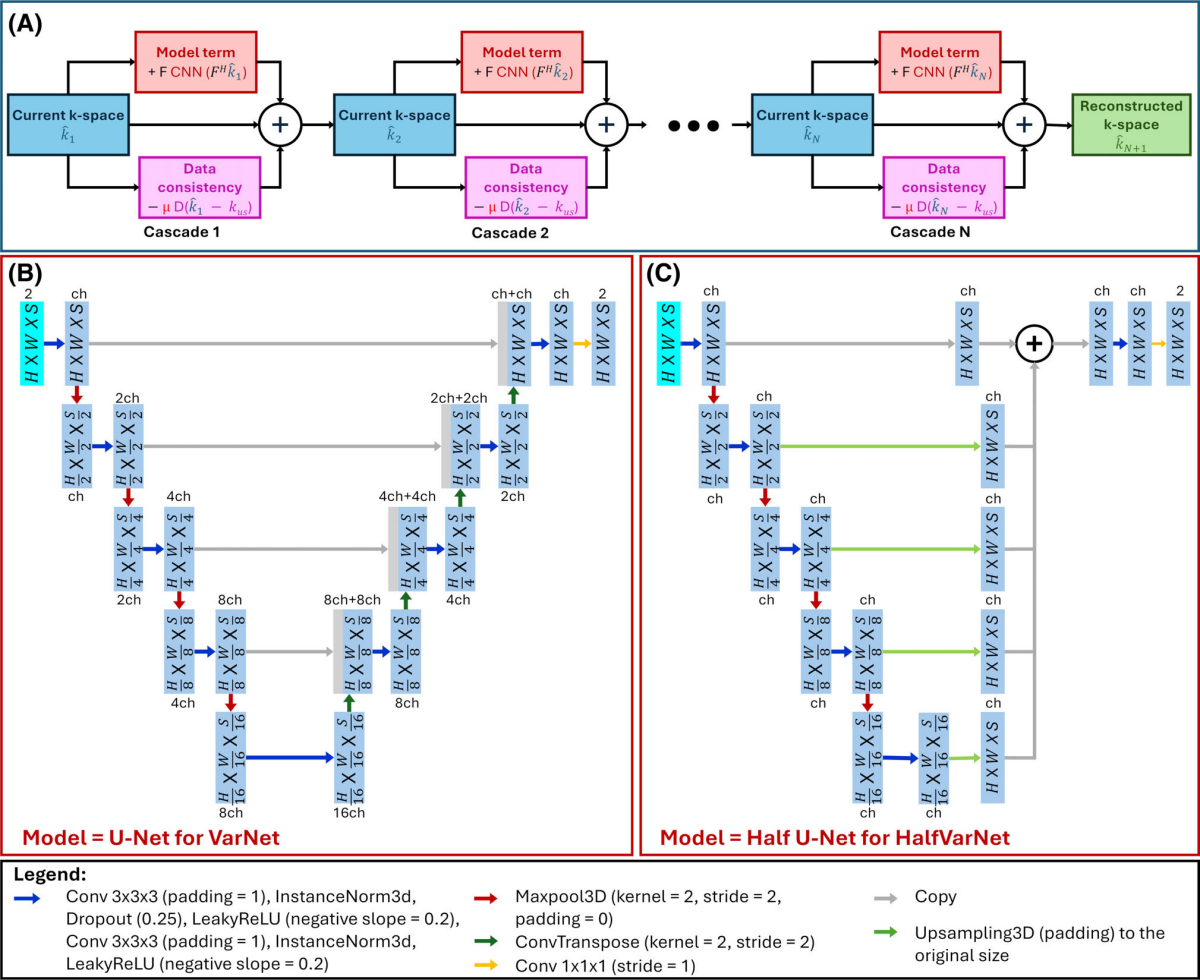
(A) represents the pipeline of VarNet. (B) U‐Net, the model used with the original VarNet. (C) Half U‐Net, the model used to obtain HalfVarNet.

VarNet uses U‐Net^17^ to estimate the gradient of the regularization function. U‐Net is a well‐known convolutional architecture initially designed for biomedical image segmentation. It consists of an encoder part and a decoder part as presented in Figure 2B. The encoder part is expected to extract important image features at different resolution levels while the decoder part learns spatial information. Skip connections between each decoder and encoder stage are useful to alleviate the vanishing gradient problem^32^ that is, to facilitate the training of the first neural network layers. At each encoder stage, the number of channels is doubled (using convolution), and the size of the image is halved (using maxpool). In the decoder part, the number of channels is halved, and the size of the image is doubled (using transconvolution) to go back to the original image size.

In the original paper, VarNet was designed for 2D multicoil data from the fastMRI^33^ dataset. In the present work, we deal with 3D single coil data. Handle 3D images required to implement a 3D U‐Net in the VarNet framework. For VarNet, the iterative gradient step is defined as

(3) $$\begin{array}{c} {\hat{k}}_{\mathrm{it}+1}={\hat{k}}_{\mathrm{it}}-\mu_{\mathrm{it}}D\left({\hat{k}}_{\mathrm{it}}-k_{\mathrm{us}}\right)+F\;\text{UNet}\left(F^{H}{\hat{k}}_{\mathrm{it}}\right) \end{array}$$

The iterative process of VarNet is presented in Figure 2A. Of interest, VarNet can be seen as cascaded blocks where the number of cascades corresponds to the number of gradient descent iterations. Each cascade *it* is composed of the data consistency term $\mu_{\mathrm{it}}\;D\left({\hat{k}}_{\mathrm{it}}-k_{\mathrm{us}}\right)$ and the model term $F\;\text{UNet}\left(F^{H}{\hat{k}}_{\mathrm{it}}\right)$. As the computation of FF maps requires reconstructing the phase of missing complex data, the input and the output of each U‐Net are here two‐channel images encoding the real and imaginary parts of the current complex image.

#### 2.2.2 DIRCN

Unrolled neural networks are generally huge networks and consequently sensitive to gradient vanishing. To address this issue, a DIRCN^18^ built on top of the architecture of VarNet was introduced. DIRCN incorporates input level dense connections that is, the input of the U‐Net in a cascade is the concatenation between the current cascade input and the inputs of all the previous cascades that is,

(4) $$\begin{array}{c} I_{\mathrm{it}}^{\text{in}}=\left[F^{H}{\hat{k}}_{1},F^{H}{\hat{k}}_{2},\ldots ,F^{H}{\hat{k}}_{\mathrm{it}}\right]. \end{array}$$

**FIGURE 3:**
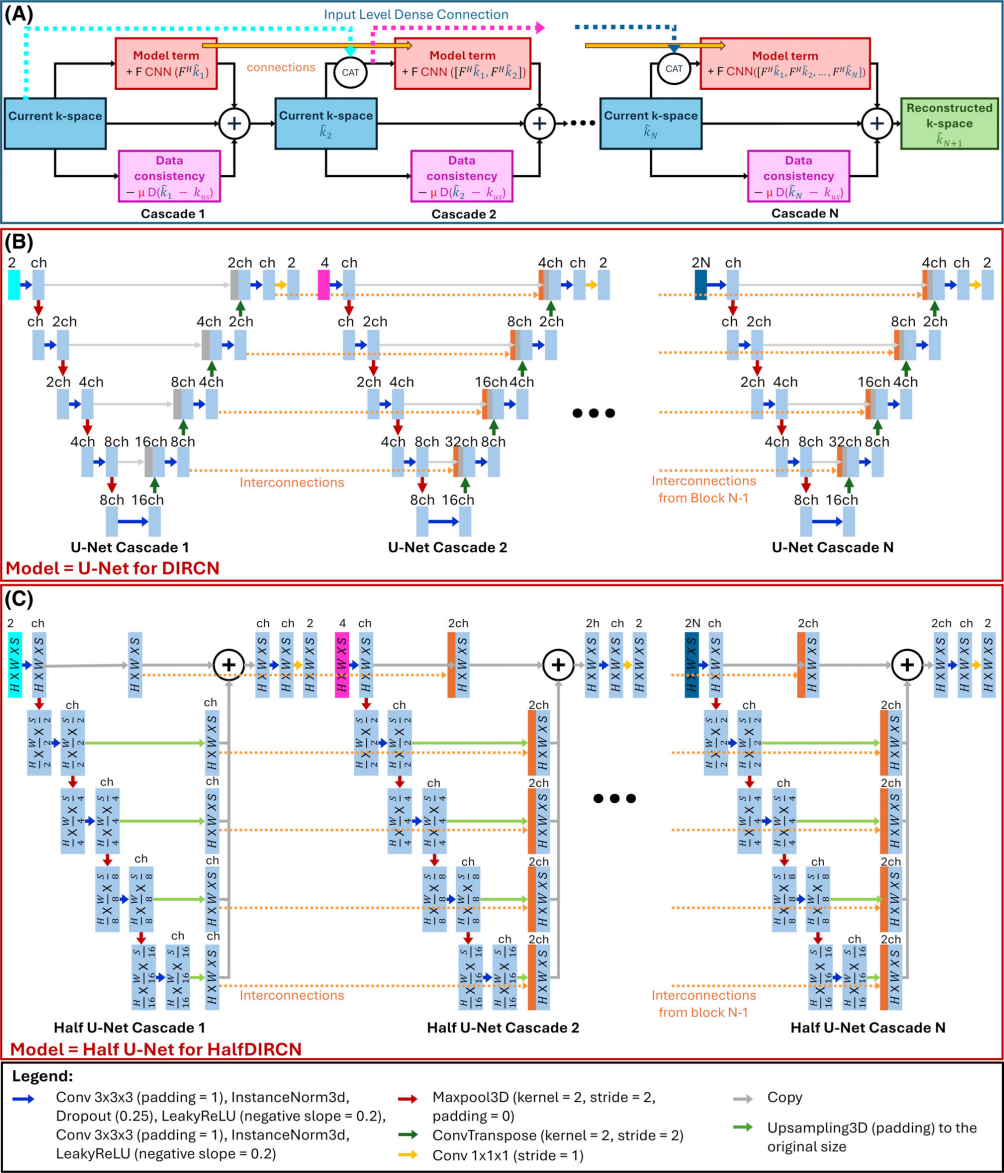
(A) The pipeline of densely interconnected residual cascading network (DIRCN). The ‘CAT’ circle denotes concatenations. (B) U‐Net, the model used inside DIRCN. The orange arrows represent the connections between each U‐Net of each cascade. (C) Half U‐Net, the model used inside HalfDIRCN.

Additionally, interconnections are incorporated between U‐Nets of two successive cascades. These long‐range skip connections are concatenations between decoder stages of the current U‐Net with the corresponding decoder stages (i.e., at the same resolution level) of the following U‐Net. DIRCN architecture is illustrated in Figure 3A,B.

### 2.3 Memory and time efficient strategy using Half U‐Net

Training deep neural networks requires high performance GPU with high memory capacity. Handling 3D data with unrolled networks may raise some limitations. While increasing the number of cascades has been shown to enhance reconstruction quality,^22^, ^24^ it can readily overwhelm the available GPU memory. The memory consumption during the training step is related to floating point operations (FLOPs). Indeed, the update of learnable parameters by backpropagation requires the storage of all operations (forward propagation) to be able to compute the gradient of the loss function. Consequently, a high number of cascades can make the computation of the gradient infeasible. In addition, prediction time naturally increases with the number of FLOPs. The number of FLOPs strongly depends on the number of convolution, maxpooling, and transpose convolution operations but also on the channel number *ch* (Figures 2 and 3).

3D U‐Nets implemented in the proposed method involve 401.26 Giga FLOPs. As a matter of example, 2D U‐Net with the same number of stages and number of input channels involves 6.86 Giga FLOPs. This significant increase clearly highlights the need for memory and time efficient modules when working with 3D data. Of interest, memory consumption depends on the batch size that is, the number of data simultaneously used to learn neural network parameters. When the number of learnable parameters is too high in comparison with the batch size, generalization performance of the networks can be deteriorated.^34^ Therefore, a trade‐off between the number of cascades and the batch size in unrolled neural network must be done. Decreasing the number of parameters in cascades is then of major importance.

Originally designed for 2D segmentation, Half U‐Net^30^ has been used for mammography, lung, and heart MRI datasets and showed a 98.6% parameter reduction while maintaining a segmentation performance comparable to U‐Net. The first simplification strategy is related to the encoder part. In U‐Net, the number of channels is doubled from one encoder stage to another while it remains constant in Half‐U‐Net. The second and the main modification is a dramatic simplification of the decoder part. Half U‐Net decoder stages only consist of upsampling the output of the corresponding encoder stage. At the last decoder stage, all the upsampled features are summed. In other words, all convolutions, transconvolutions, LeakyReLU, maxpooling, and instance normalization operations are removed from the decoder (except in the last decoder stage). 3D Half U‐Net involves 146.39 Giga FLOPs that is approximately three times fewer FLOPs than 3D U‐Net. The proposed approach consists in using Half U‐Net as model term in VarNet and DIRCN. Both novel networks, referred to as HalfVarNet and HalfDIRCN, are presented in Figures 2C and 3C, respectively. For HalfDIRCN, the decoding part (after the upsampling and before the addition) to each stage of two successive Half U‐Nets are interconnected. Since using Half U‐Net reduces memory consumption and learnable parameters, HalfVarNet and HalfDIRCN offer the possibility to incorporate more cascades than the regular VarNet and DIRCN.

### 2.4 Dataset

#### 2.4.1 Dixon images

Patients were positioned supine within the MRI scanner, and the non‐dominant thigh was imaged using a flexible coil on the top and spine coils on the bottom (integrated into the scanner bed). MRI acquisitions were performed at 1.5T clinical (MAGNETOM Avanto, Siemens Healthcare GmbH, Erlangen, Germany). A 3D chemical‐shift‐based water‐fat separation gradient recalled echo sequence was used with the following parameters: TR = 22 ms, TE = 2.38 to 19.06 ms with 2.38 ms step, flip angle = 5°, matrix size = 128 × 128 × 36, resolution = 1.72 × 1.72 × 5.00 mm^3^. Each acquisition consisted of eight echoes. The acquisition time was 3 min 24 s. Acquisitions were performed with a eight‐channel coil compressed into one‐channel data using the vendor algorithm. The magnitude and the phase of the echo were stored. Consequently, in the present study, data at our disposal are single coil complex‐valued k‐spaces. Undersampling was performed retrospectively by applying a variable density Poisson disk undersampling^35^ with a fully sampled center of 4% of the k‐space. The undersampling was performed for an acceleration factor of 4 and 8.

#### 2.4.2 Subjects

The study was approved by the local research committee and was conducted in conformity with the Declaration of Helsinki (version October 2013) and the Medical Research Involving Human Subjects Act. A written informed consent was obtained from each subject regarding their participation in the project and the retrospective analysis of the MR images. Data were collected as part of previous studies conducted in the Reference Center of neuromuscular disorders and ALS in la Timone hospital and dedicated to three types of diseases that is, Charcot Marie Tooth (CMT1A), chronic inflammatory demyelinating polyneuropathy (CIDP), and familial amyloid polyneuropathy (FAP). MRI acquisitions were performed in 134 subjects that is, 13 controls, 43 CMT patients, 28 CIDP patients, and 50 FAP patients (age: 47 ± 16 y (range 17–83 y), sex: 63F and 84 M, weight: 69 ± 14 kg). CMT is the most common form of inherited neuropathy.^25^ CMT type 1 is the demyelinating form of the disease. CMT1A is the most common form affecting 50% of patients with CMT, and it is caused by a mutation of the PMP22 gene. CIDP is an autoimmune neuropathy affecting peripheral nerve and nerve root function.^36^ Additionally, FAP is a rare disorder where nerve lesions are caused by deposits of amyloid fibrils, resulting from mutated transthyretin (TTR), and causes a rapid progressive polyneuropathy.^37^ The control group was composed of individuals with no medical history of neuromuscular pathology. For each MRI dataset, FF values were computed using the first three echoes, as suggested by Glover et al.^38^ for each individual muscle of the thigh.

### 2.5 Model training

**FIGURE 4:**
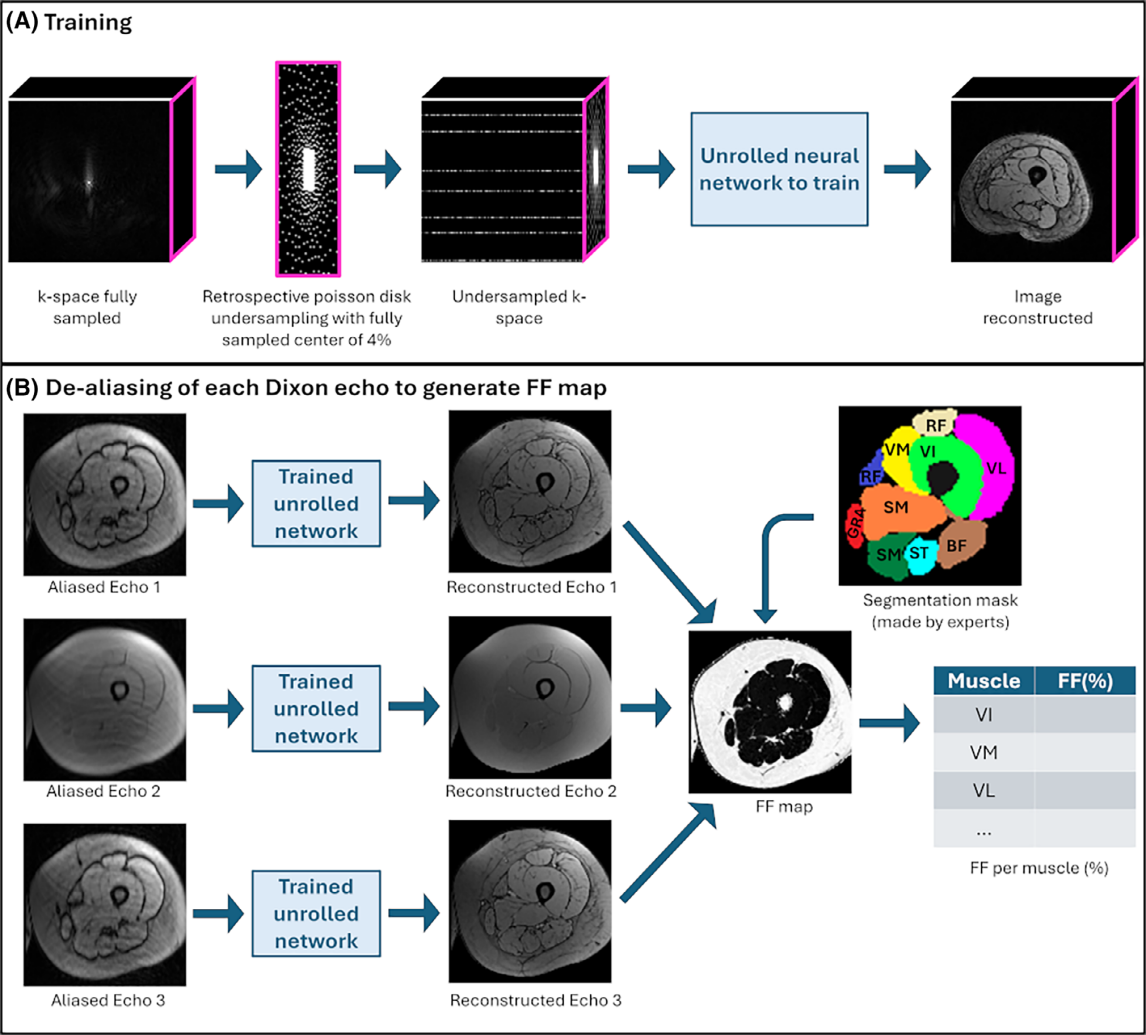
(A) Training pipeline. Acquisitions are retrospectively undersampled using a poisson disk undersampling. Then the undersampled k‐space is used to train the unrolled networks (VarNet, HalfVarNet, DIRCN or HalfDIRCN). (B) The prediction pipeline to generate FF maps. Each aliased echo is reconstructed using an unrolled network. First three echoes are combined to generate the FF map. Then the mask associated with echo 1 is propagated to the FF map to compute the FF for each muscle.

Preliminary results have shown that 8 cascades with a batch size of four 3D images reached high reconstruction performance on our dataset using VarNet and DIRCN. Accordingly, we performed a comparison study using HalfVarNet and HalfDIRCN with eight cascades. Additionally, HalfVarNet and HalfDIRCN with 12 cascades were added to the study. The whole dataset contained 180 Dixon acquisitions, each with eight echoes. Each Dixon acquisition was split up echo by echo leading to a total of 1440 volumes. Some patients did multiple acquisitions which explained why the number of acquisitions exceeded the number of patients. The dataset was split between training (1112 volumes), validation (168 volumes), and test (160 volumes). The training dataset included 139 acquisitions from 102 subjects with 37 patients scanned twice at least 1 y apart. The validation dataset consisted of 21 acquisitions from 16 subjects including 5 patients with two scans at least 1 y apart. The test dataset included 20 acquisitions from 16 subjects with 4 patients scanned twice over a year interval. To avoid reconstruction bias, all echoes from a single acquisition were assigned to the same dataset. Similarly, images corresponding to a subject (patient or control) were not split between the datasets. For training step, retrospectively undersampled k‐space were used as input and passed through the unrolled networks. Each architecture was trained twice: once for the acceleration factor 4 and once for the acceleration factor 8. The training pipeline is presented in Figure 4A. All methods were trained on a linux station using one Nvidia A100D‐80C with a python environment and PyTorch 1.13.1. The complex k‐space values were split into two channels (real and imaginary parts) and normalized with a mean of 0 and a SD of 1 before going into the U‐Nets or Half U‐Nets. The number of channels (ch variable in Figures 2 and 3) was set to 32, the number of stages was set to five (four maxpooling layers), and the dropout rate was 0.25. The Adam optimizer was used with 0.001 learning rate. The MSE loss was computed on the inverse Fourier transform of the predicted k‐space and on the fully sampled reference image. A maximum number of 500 epochs were used. The selected models were those who achieved the lowest validation loss during the training step.

### 2.6 Evaluation strategy

#### 2.6.1 Reconstruction performance

The performance of each unrolled network was evaluated on the test dataset on FF map generated from the first three echoes and compared to the FF map generated with the fully sampled data. Image background was removed before evaluation. Reconstruction performance was assessed by providing average values and the standard errors (computed over the test dataset) of three metrics: the mean square error (MSE), the structural similarity index (SSIM), and the peak SNR (PSNR). The assessment of the average FF in each individual muscle is crucial for the follow‐up of NMD. The regions of interest (ROIs) were adductor (ADD), semimembranosus (SM), semitendinosus (ST), biceps femoris (BF), vastus intermedius (VI), gracilis (GRA), sartorius (SAR), vastus medialis (VM), rectus femoris (RF), and vastus lateralis (VL). Muscle segmentation was performed by a neurologist with more than 5‐y experience of segmentation. Segmentation was carried out in a small number of slices, and a semi‐automatic method was used to propagate the corresponding masks to the remaining slices.^39^ Segmentation results were double‐checked by experts and corrected if necessary. The corresponding masks created on anatomical images were registered and resliced to the corresponding first Dixon echo and FF map to assess the FF value for each muscle. The reconstruction process and ROI are presented in Figure 4B. The FF was evaluated for each individual muscle using the FF relative quadratic error (denoted ${\mathrm{FF}}_{\text{quadratic}}$ error) between average FF value of the considered muscle in the fully sampled data and the reconstructed data. The absolute mean difference was also computed for comparison with the 12‐month variation follow‐up in NMDs.

#### 2.6.2 Delimitation of ROI

Accurate FF measurement requires a precise muscle delineation. Accordingly, it is important to determine whether the reconstructed images have sufficient quality to delineate each muscle. The delineation should be done on the first out‐of‐phase echo because it provided superior definition of muscle borders compared to other echoes. The manual segmentation of individual muscles is tedious, time‐consuming, and prone to errors among different operators. To address this issue, numerous automatic methods have been proposed. In the present study, we chose nnU‐Net,^31^ a state‐of‐the‐art segmentation network, trained on our fully sampled data to generate individual muscle masks. The segmentation provided by nnU‐Net was compared to the reference mask made by experts using Dice similarity coefficient (DSC), sensitivity (true positive rate), specificity (true negative rate), and ${\mathrm{FF}}_{\text{quadratic}}$ segmentation error. For a given image (fully sampled or reconstructed), average FF value in an individual muscle was assessed by applying the nnU‐Net segmentation mask and the expert segmentation mask. ${\mathrm{FF}}_{\text{quadratic}}$ segmentation error was then computed by performing the relative quadratic error between both previous results.

## 3 RESULTS

### 3.1 Reconstruction metrics

**FIGURE 5:**
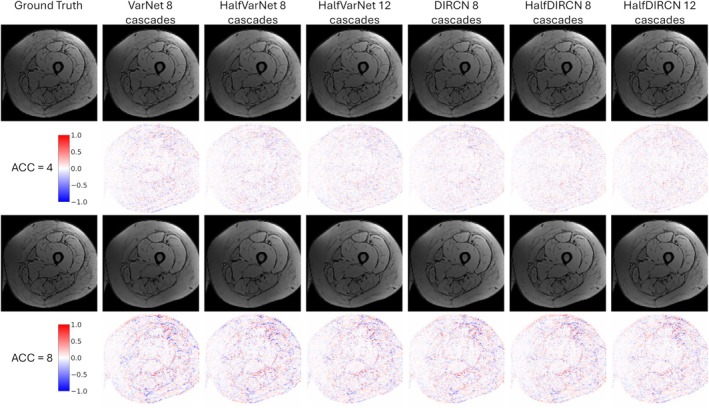
Visual representation of the results for the six networks tested and for both acceleration factors. This example is a out‐of‐phase Dixon echo. Second line and fourth line represent the difference between the ground truth and the reconstructed images.

**FIGURE 6:**
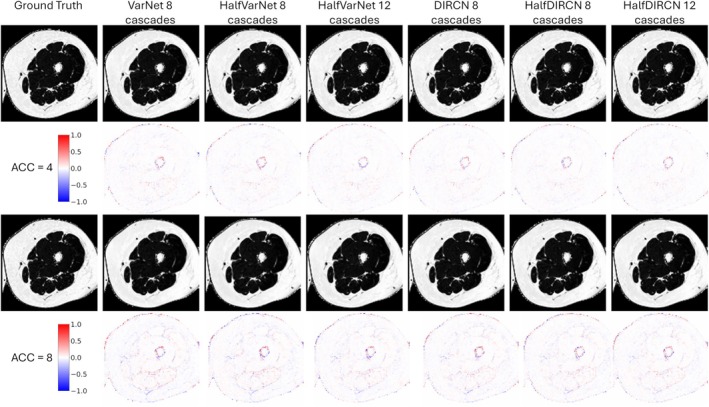
Visual representation of the results for the 6 networks tested and for both acceleration factors. This example is a FF map. Second line and fourth line represent the difference between the ground truth and the reconstructed image.

Reconstruction results are summarized in Table 1 with localization of errors presented in Figure 5 for the first out‐of‐phase echo and in Figure 6 for the FF map. These figures display the differences between the fully sampled data and the reconstructed data. Largest errors occurred at the borders of the thigh and the borders between muscles.

**TABLE 1:** Reconstruction performance for the acceleration factor of 4 and the acceleration factor of 8.

Acceleration factor	Reconstruction method	MSE (×10^−2^)	SSIM (%)	PSNR	$\text{Mean}\;{\mathrm{FF}}_{\text{quadratic}}$ error (%)
**4**	VarNet 8 cascades	6.68 ± 0.57	96.66 ± 0.26	52.34 ± 0.66	0.17 ± 0.02
	HalfVarNet 8 cascades	6.40 ± 0.54	96.76 ± 0.25	52.46 ± 0.60	0.10 ± 0.01
	HalfVarNet 12 cascades	5.77 ± 0.48	97.09 ± 0.24	52.90 ± 0.60	**0.08 ± 0.01**
	DIRCN 8 cascades	5.42 ± 0.47	97.34 ± 0.21	53.25 ± 0.65	0.09 ± 0.01
	HalfDIRCN 8 cascades	5.46 ± 0.48	97.32 ± 0.22	53.20 ± 0.63	0.08 ± 0.01
	HalfDIRCN 12 cascades	**5.25 ± 0.45**	**97.41 ± 0.21**	**53.36 ± 0.63**	0.08 ± 0.01
**8**	VarNet 8 cascades	13.13 ± 1.15	93.59 ± 0.40	49.49 ± 0.71	0.51 ± 0.05
	HalfVarNet 8 cascades	12.76 ± 1.08	93.76 ± 0.38	49.49 ± 0.62	0.34 ± 0.04
	HalfVarNet 12 cascades	10.72 ± 0.91	94.70 ± 0.34	50.28 ± 0.64	0.23 ± 0.02
	DIRCN 8 cascades	10.50 ± 0.93	94.82 ± 0.34	50.45 ± 0.70	0.30 ± 0.03
	HalfDIRCN 8 cascades	10.25 ± 0.87	94.95 ± 0.32	50.50 ± 0.67	**0.17 ± 0.02**
	HalfDIRCN 12 cascades	**9.76 ± 0.86**	**95.19 ± 0.32**	**50.72 ± 0.67**	0.22 ± 0.02

*Note*: Bold values represent the best performance.

Reconstruction performance was obviously superior for an acceleration factor of 4 compared to a factor of 8. DIRCN outperformed VarNet across all reconstruction metrics. HalfVarNet with 8 cascades improved results compared to VarNet for both acceleration factors but did not outperform the performance of DIRCN. Adding more cascades (HalfVarNet 12 cascades) further improved reconstruction performance. HalfDIRCN 8 cascades improved results for the acceleration factor of 8. HalfDIRCN 12 cascades achieved the best reconstruction performance for both acceleration factors.

Concerning FF computation, the lowest ${\mathrm{FF}}_{\text{quadratic}}$ error for an acceleration factor of 4 was achieved with HalfVarNet 12 cascades, HalfDIRCN 8 and 12 cascades. For factor 8, the lowest ${\mathrm{FF}}_{\text{quadratic}}$ error was achieved with HalfDIRCN 8 cascades. Interestingly, ${\mathrm{FF}}_{\text{quadratic}}$ error was not completely correlated to the reconstruction performance.

### 3.2 Delimitation of ROI and biomarker assessment

Segmentation results are summarized in Table 2. Considering the whole set of muscles automatically delineated using nnU‐Net trained on the fully sampled data, the average DSC score was 92.83 ± 0.19% and the mean ${\mathrm{FF}}_{\text{quadratic}}$ error was 2.13 ± 0.25% on the test set. For the whole set of muscles segmented using nnU‐Net trained on the reconstructed data, the average DSC ranged from 92.78 ± 0.19 to 92.84 ± 0.19% for the acceleration factor of 4 and between 92.56 ± 0.20 and 92.82 ± 0.19% for the acceleration factor of 8. The closest average DSC to the fully sampled reference was the one obtained using HalfDIRCN 12 cascades (92.83 ± 0.19% for the acceleration factor of 4 and 92.82 ± 0.19% for the acceleration factor of 8) and corresponded to the best segmentation performance. In contrast, the lowest DSC was obtained with VarNet 8 cascades for both acceleration factors. DSC results showed that similar segmentation results can be reached using fully sampled data or accelerated acquisitions. The sensitivity and the specificity were also evaluated. For segmentation on fully sampled data, specificity was 99.85 ± 0.11% and sensitivity as 93.86 ± 3.52%. Across all reconstruction methods and for both acceleration factors, sensitivity values ranged from 93.62 ± 3.76 to 93.83 ± 3.59%, indicating that the segmental models detected the majority of the muscles to be classified, with only a small variation across methods. Interestingly, specificity remains equal to 99.85% for all reconstruction methods. This suggests that the models avoid false positives (ignore regions that do not contain the muscle to segment) regardless of the reconstruction method used. Regarding FF estimation, the results could suggest that estimating FF in individual muscles with accelerated acquisition was more efficient than using fully sampled data. These results should be considered with caution and will be explained in the discussion part.

**TABLE 2:** Segmentation performance for the acceleration factor of 4 and the acceleration factor of 8.

Acceleration factor	Reconstruction method	Mean DSC (%)	Specificity	Sensitivity	Mean ${\mathrm{FF}}_{\text{quadratic}}$ segmentation error (%)
Fully sampled		92.83 ± 0.19	99.85 ± 0.11	93.86 ± 3.52	2.13 ± 0.25
**4**	VarNet 8 cascades	92.78 ± 0.19	99.85 ± 0.11	93.83 ± 3.59	1.96 ± 0,22
	HalfVarNet 8 cascades	92.81 ± 0.19	99.85 ± 0.11	93.81 ± 3.58	1.95 ± 0.22
	HalfVarNet 12 cascades	92.81 ± 0.20	99.85 ± 0.11	93.80 ± 3.63	1.94 ± 0.22
	DIRCN 8 cascades	92.84 ± 0.20	99.85 ± 0.11	93.82 ± 3.62	1.95 ± 0.22
	HalfDIRCN 8 cascades	92.84 ± 0.19	99.85 ± 0.11	93.78 ± 3.63	2.01 ± 0.23
	HalfDIRCN 12 cascades	92.83 ± 0.19	99.85 ± 0.11	93.78 ± 3.63	1.98 ± 0.23
**8**	VarNet 8 cascades	92.56 ± 0.20	99.85 ± 0.11	93.75 ± 3.65	1.88 ± 0.21
	HalfVarNet 8 cascades	92.58 ± 0.20	99.85 ± 0.11	93.62 ± 3.76	1.89 ± 0.21
	HalfVarNet 12 cascades	92.75 ± 0.20	99.85 ± 0.11	93.75 ± 3.65	1.87 ± 0.21
	DIRCN 8 cascades	92.70 ± 0.20	99.85 ± 0.11	93.73 ± 3.68	1.97 ± 0.22
	HalfDIRCN 8 cascades	92.74 ± 0.20	99.85 ± 0.11	93.73 ± 3.65	1.97 ± 0.22
	HalfDIRCN 12 cascades	92.82± 0.19	99.85 ± 0.11	93.82 ± 3.60	1.87 ± 0.21

*Note*: nnU‐Net was trained on fully sampled data. nnU‐Net was trained on fully sampled data. DSC was computed between the masks obtained using nnU‐Net and masks made by experts. ${\mathrm{FF}}_{\text{quadratic}}$ segmentation error was computed as the relative quadratic error between the average FF in individual muscles obtained from nnU‐Net segmentation and the average FF in individual muscles obtained from expert segmentation masks.

### 3.3 Model performance

**FIGURE 7:**
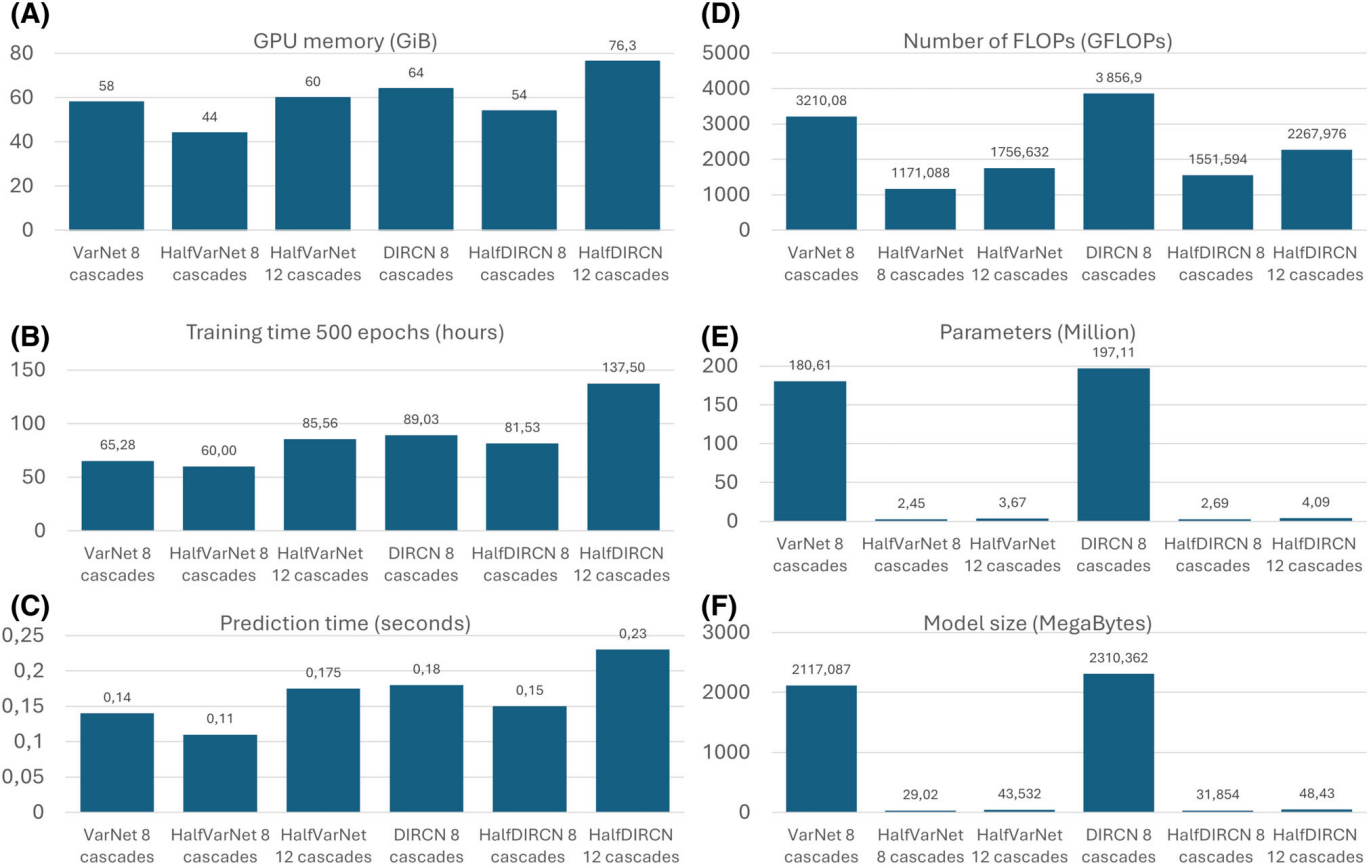
Memory (A), training time (B), prediction time (C), number of FLOPs (D), parameters (E), and model size (F) for the six different architectures tested.

Figure 7A highlights the memory efficiency of the “Half” networks. Using eight cascades, Half versions reduce the memory usage compared to VarNet 8 cascades and DIRCN 8 cascades, decreasing from 58 GiB to 44 GiB and from 64 GiB to 54 GiB, respectively. Half versions with 12 cascades use more GPU memory than the original VarNet and DIRCN: 60 GiB for HalfVarNet 12 cascades and 76 GiB for HalfDIRCN 12 cascades. Interestingly, HalfVarNet 12 cascades used almost the same memory as VarNet 8 cascades while HalfDIRCN 12 cascades outperformed dramatically the memory usage of DIRCN 8 cascades. In Figure 7B,C, the training time and prediction time were correlated with the GPU memory usage. HalfDIRCN 12 cascades had the largest training time (137.5 h for 500 epochs) and testing time (0.11 s) while the lowest training time (60 h for 500 epochs) and prediction time (0.23 s) were obtained with HalfVarNet 8 cascades. Figure 7D–F displays the number of FLOPs, the number of parameters, and model size in megabytes (MB). The three features were reduced using Half networks.

For VarNet, the number of FLOPs, parameters, and model size were, respectively, 3210 GFLOPs, 180 million parameters, and 2.1GB. It was reduced to 1171 GFLOPs, 2.45 million of parameters, and 29 MB for HalfVarNet 8 cascades and to 1756 GFLOPs, 3.67 million parameters, and 44 MB for HalfVarNet 12 cascades. For DIRCN, the number of FLOPs, parameters, and model size were, respectively, 3856 GFLOPs, 197 million parameters, and 2.3 GB. It was reduced to 1551 GFLOPs, 2.69 million parameters, and 32 MB for HalfDIRCN 8 cascades and to 2268 GFLOPs, 4.09 million parameters, and 48 MB for HalfDIRCN 12 cascades.

## 4 DISCUSSION

DIRCN provided better reconstruction results than VarNet at the cost of a higher GPU memory utilization, a longer training/prediction times, a higher number of FLOPs, and higher model size. With the same cascade number, the proposed ‘Half’ approach led to a general improvement in reconstruction metrics. Reconstruction quality can be further improved by increasing the number of cascades. Of interest, at a given acceleration factor, Half VarNet was never able to outperform Half DIRCN regardless of the number of cascades.

The biases of FF in individual muscles for reconstructed images are ranged from 0.18% to 0.44% with corresponding 95% limits of agreements between 0.35% and 0.98%. These limits indicate low additional random uncertainty during the reconstruction process and support the reliability of the results. Moreover, the measured biases were lower than the reported rate of disease progression that is, 1.04%/y over a 12 mo follow‐up in CMT1A patients.^40^

nnU‐Net trained on the fully sampled dataset achieved 92.83 ± 0.19% of DSC value on fully sampled test dataset. Of interest, this performance was similar regardless of the test dataset that is, fully sampled or reconstructed with unrolled networks. Interestingly, the better the reconstruction metrics, the higher the DSC value. ${\mathrm{FF}}_{\text{quadratic}}$ error (using expert segmentation masks) was largely smaller than the ${\mathrm{FF}}_{\text{quadratic}}$ segmentation error (using nnU‐Net segmentation masks). These results indicate that FF measurements are more sensitive to segmentation errors than to reconstruction errors. Moreover, in a previous work,^41^ we showed that the segmentation performance of neural networks and quality of FF assessment can be strongly uncorrelated. Our results illustrate that reconstruction methods provide very similar results as compared to the fully sampled dataset in terms of FF measurements. If one intends to use unrolled neural network for the fully automatic follow‐up of NMD, the preferred choice should be Half VarNet 8 since it provided similar results with the fully sampled and the accelerated datasets, whereas the computing resources needed were substantially reduced. Indeed, it had the lowest GPU memory utilization, the shortest training/prediction time, the lowest number of FLOPs, and the lowest model size.

Ablation experiments on Half U‐Net architecure were conducted before running all tests. For upsampling, the transpose convolution method was compared to PyTorch upsampling methods namely the “nearest” and the “trilinear” modes. While reconstruction performance was similar across all methods, the trilinear upsampling method achieved slightly better reconstruction metrics and reduced the number of parameters in HalfVarNet by a factor of 20 compared to the version using transpose convolutions. Based on these results, the “trilinear” upsampling method was selected for HalfVarNet and HalfDIRCN.

Wang et al.^22^ as well as Miller et al.^24^ showed that increasing the number of cascades could improve the results. If needed, ‘Half’ versions can be used with more cascades with the goal of improving reconstruction results. Accordingly, we were able to use HalfVarNet 12 cascades and HalfDIRCN 12 cascades, which would not be possible with the original VarNet and DIRCN considering the available computing resources. While increasing the number of cascades comes at the cost of a higher memory usage and training time, it improves reconstruction performance. Additionally, in the proposed approach, the number of parameters and the model size was divided by approximately 50 compared to their original versions which is advantageous if one intends to deploy the model on resources‐constrained devices.

Deep learning reconstruction approaches have been previously developed for high‐dimensional images such as 4D (space + time) MRI. Freedman et al.^42^ proposed to train 3D U‐Nets suited to the online treatment adaptation in thoracic or abdominal MR‐guided radiotherapy. Hauptmann et al.^43^ developed a residual U‐Net like architecture to reconstruct real time cardiovascular spatio‐temporal MR images in the context of congenital heart disease. Authors have shown that their method enabled a five times faster reconstruction as compared to compressed sensing with no statistically significant difference from the gold standard. In our case, preliminary works showed that unrolled neural networks outperformed U‐Net and classical compressed sensing algorithms for the reconstruction of Dixon acquisitions.

Sandino et al.^44^ used an unrolled neural network for the reconstruction of multi‐slice 2D cardiac cine MRI data from multi‐coil acquisition. Sensitivity maps were derived using ESPIRIT.^29^ Each 4D data (kx, ky, slice, cardiac phase) was split up slice by slice to increase the number of training examples while reducing their dimension. In our case, each Dixon acquisition (kx, ky, kz, echo) was split up echo by echo for the same reasons. Of interest, if a fully sampled Dixon is acquired, 3D Fourier transforms applied on each echo separately are sufficient to reconstruct the multi‐echo images.

### 4.1 Limitations and future work

One has to acknowledge several limitations in the present study. The present experiment was conducted on a dataset equivalent to single‐coil acquisition although it was acquired with multi‐coil imaging which could introduce bias during reconstruction with neural network. The undersampling was done retrospectively on these images. HalfVarNet and HalfDIRCN should be applied in more practical scenarios closer to reality. However, these results demonstrate great potential for deep learning in MRI reconstruction. Furthermore, the code is fully transferable to multi‐coil imaging since HalfVarNet and HalfDIRCN are based on VarNet and DIRCN, which are already compatible with multi‐coil data.

Patients with FF values ranging from 4.43% to 82.65% were included with the majority of muscles having a FF value ranging below 20% (90% of values below 20%) and a median value of 8.91%. It has been reported in dystrophic patients that muscles with intermediate values of FF that is, between 20% and 50% displayed the fastest disease progression, a phase during which potential treatment would be more effective. In that respect, it would be of interest in future studies to assess whether Half models would still be effective for patients with larger infiltration.

Previous studies on high‐dimensional MRI reconstruction could be interesting for future investigations.

For instance, Küstner et al.^45^ proposed an unrolled neural network using complex convolutions for the reconstruction of multi‐coil MRI.

Murray et al.^46^ accelerated the acquisition of golden‐angle radial data by exploiting the space–time coil correlations and motion preservation. It could be interesting to investigate if correlation between echoes can improve the reconstruction. Multi‐echo reconstruction would directly lead to an increased memory utilization and to a decreased batch size. It would then be necessary to find the best trade‐off.

Xu et al.^47^ proposed an unrolled neural network composed of two parallel branches for image and k‐space domains. The image branch allowed to enforce the sparsity and the local low rank property of dynamic MR images while sharing useful information from the k‐space branch using attention mechanisms. At each cascade, a “low‐rank subnetwork” was dedicated to extract local spatio‐temporal relationships. The input image sequence is consequently split into spatio‐temporal patches which decreases the computational cost. The proposed network can efficiently reconstruct highly retrospectively undersampled dynamic MR images up to an acceleration factor of 24.

## 5 CONCLUSIONS

This paper introduces two novel unrolled networks, HalfVarNet and HalfDRICN, using Half U‐Net instead of the traditional U‐Net. Half models were trained and tested on a 3D Dixon database of thigh muscles from NMD patients and enabled to reduce memory usage, training time, and prediction time in comparison with VarNet and DIRCN. The reconstruction performance was slightly improved overall. Regarding the FF estimation, errors were marginally smaller with the proposed approach. Increasing the number of cascades with VarNet and DIRCN is limited by GPU constraints. While this increase is made possible with Half versions and leads to the improvement in performance.

Finally, a neural network dedicated to segmentation (used to compute FF values in each muscle) was trained on the fully sampled data to enable automatic segmentation. Results show that reconstructed images can be properly segmented. Indeed, DSC scores were similar to those obtained from the fully sampled dataset. Computation of FF in individual muscles with the proposed approach also provided similar results as those reached with fully sampled data. Thus, our results showed that MRI acquisition can be strongly accelerated for the follow‐up of patients with neuromuscular disorders without the requirement for high computing resources.

## CONFLICT OF INTEREST STATEMENT

Sandra Martin and Amira Trabelsi are both employed by Multiwave Technologies SAS.

## Data Availability

The data that support the findings of this study are available from the corresponding author upon reasonable request. The implementation of the models is available in the following repository: https://gitlab.com/sandra_martin/half_public.
